# Learning Needs of Dementia Family Caregivers during the Pandemic

**DOI:** 10.1093/geroni/igab046.2948

**Published:** 2021-12-17

**Authors:** Carolyn Clevenger, Fayron Epps, Molly Perkins, Glenna Brewster Glasgow, Kenneth Hepburn

**Affiliations:** 1 Emory University, Emory University, Georgia, United States; 2 Emory University, Atlanta, Georgia, United States; 3 Emory University of Medicine, Atlanta, Georgia, United States

## Abstract

The COVID-19 pandemic has dominated and transformed all caregiving contexts and situations. In a time of COVID-19, caregivers now have to learn how to take all of the complicated precautions to keep themselves and their persons from being exposed to the virus given their population’s mortality rate from COVID-19 infections exceed 40%. As part of a larger initiative to develop an asynchronous online education program for family caregivers of persons living with dementia illnesses (PLWD) to prepare them to master the new demands of their caregiving role in this extraordinary circumstance of the COVID-19 pandemic, we conducted three focus groups with 13 dementia family caregivers to inform the structure, content, and “feel” of the course. Focus groups were conducted with a lead interviewer, via Zoom, audio and video recorded and transcribed for analysis. Participants were asked two groups of questions: their lived experience over the past year and course content for caregiving during crisis. Caregivers identified 4 themes regarding their lived experience of caregiving during the pandemic: (a) mixed feeling about the stay-at-home orders; (b) positive adaptation to telemedicine, (c) vaccine risks and benefits; and (d) impact of social isolation on the PLWD. The groups also recommended specific course content based on their experiences. Participants recommended specific course content based on their experiences, such as health system navigation and the logistics of what to do following the death of a PLWD. Results from these groups have been incorporated into an asynchronous online course to be pilot tested in coming months.

